# Beta_2_-glycoprotein I inhibition of mouse Kupffer cells respiratory burst depends on liver architecture

**DOI:** 10.1186/1476-5926-2-S1-S43

**Published:** 2004-01-14

**Authors:** Ligia F Gomes, Paula R Knox, Karin A Simon-Giavarotti, Virginia BC Junqueira, Jorge Sans, Luis A Videla

**Affiliations:** 1 Departamento de Análises Clánicas e Toxicolágicas, Faculdade de Ciáncias Farmacáuticas, Universidade de Sáo Paulo, Sáo Paulo, Brasil; 2 Disciplina de Geriatria, Departamento de Medicina, Universidade Federal de Sáo Paulo, Sáo Paulo, Brasil; 3 Unidad de Análisis Celular Integral, ICBM, Facultad de Medicina, Universidad de Chile, Santiago, Chile; 4 Programa de Farmacologáa Molecular y Clánica, ICBM, Facultad de Medicina, Universidad de Chile, Santiago, Chile

## Introduction

Kupffer cells play important roles in the modulation of immune response, phagocytosis, and senescent cell removal [1,2]. Hydrolytic enzymes and reactive species produce the killing effects of Kupffer cells and some degree of adjacent tissue damage [1,3]. Liver macrophages are constantly exposed to antigens from portal circulation, to which development of full inflammatory response is useless and potentially harmful [4]. Neither tissue damage nor inflammation follows senescent cell removal by Kupffer cells, due to the physiological control of inflammation events during antigen processing [2,5]. Apolipoproteins can modulate macrophage function [6]. Among them, beta_2_-glycoprotein I (beta_2_GPI) decreases Kupffer cells respiratory burst while increases efficiency of *C. albicans *killing [7]. Beta_2_GPI also binds phosphatidylserine (PS) residues on the surface of senescent cells, targeting them to clearance [8]. In order to get an insight on the role of beta_2_GPI in the silent antigen removal by Kupffer cells, perfused mouse liver was used as a model of Kupffer cell-dependent phagocytosis and related respiratory burst activity, and results were correlated with those obtained in isolated mouse non-parenchymal cells.

## Methods

All reagents used were obtained from Sigma (St. Louis, MO), except for beta_2_GPI that was purified from a pool of human sera [7]. Livers from female CF-1 mice (20á28 g body weight) fed *ad libitum *were perfused with Krebs-Henseleit bicarbonate buffer pH 7.4, saturated with 95% O_2_/5% CO_2_, at 10 mL/min and 37 degrees C, without recirculation [9]. After 15 min equilibration, O_2 _uptake was measured in the effluent perfusate as it flowed past a Clark-type O_2 _electrode. Total sinusoidal lactate dehydrogenase (LDH) efflux (in U/g liver) and the respective fractional LDH release (in % of the activity in the tissue) were assessed in the 30á45 min interval as described [9]. Colloidal carbon (C) (0.25 mg/mL; Rotring, Germany) was infused during the 30á45 min interval, either in the absence or presence of 1á30 micrograms beta_2_GPI/mL, added at 20 min, and rates of C uptake were calculated according to Cowper et al. [10]. Carbon-induced O_2 _consumption (in micromolar O_2_/g liver/min) was calculated by subtracting the basal O_2 _uptake, during the 30á45 min C perfusion interval [9]. Liver samples taken after perfusion with 0.25 mg C/mL in the absence and in the presence of 30 micrograms beta_2_GPI were fixed in Dubosq Brazil, embedded in Paraplast, and stained with hematoxylin-eosin. Non-parenchymal liver cell preparation was obtained by liver perfusion with collagenase [7], with viability values higher than 95%. The respiratory burst was evaluated by a luminol-dependent assay [11] after zymosan stimulation (200 particles/cell) [7], and results were expressed as relative total light emission or light emission rate.

## Results and Discussion

**Figure 1:**
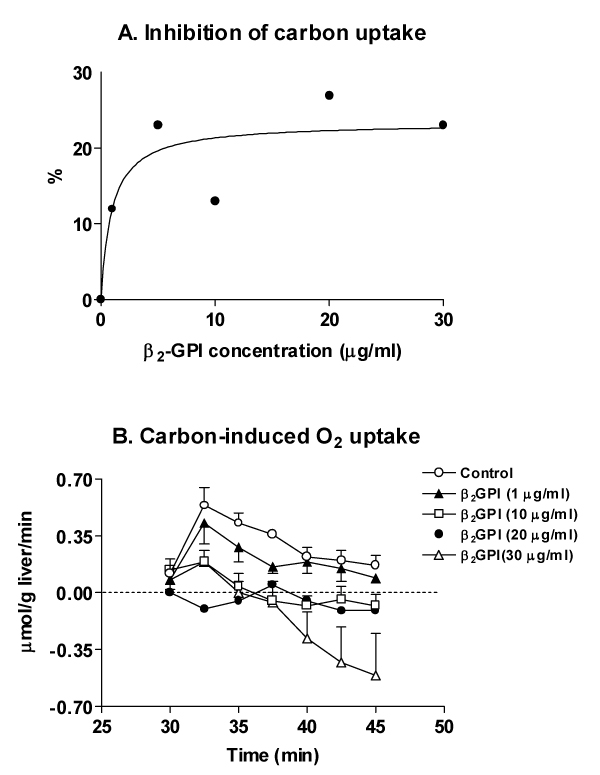
Infused beta_2_-glycoprotein I effects on the perfused mouse liver (A) C-uptake and (B) C-induced O_2 _consumption. Means á SEM for three to five animals/group.

**Figure 2:**
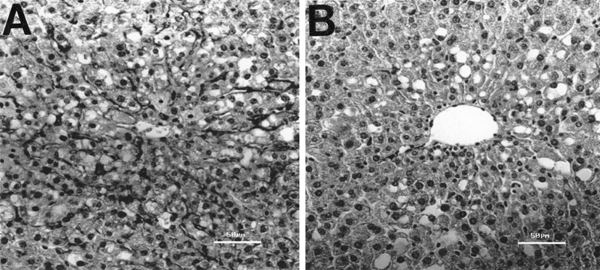
Structural characteristics of mouse liver parenchyma perfused in vitro with 0.25 mg of colloidal carbon/mL in the (A) absence and (B) presence of 30 micrograms /mL of beta_2_-glycoprotein I (beta_2_GPI). Haematoxylin-eosin.

**Figure 3:**
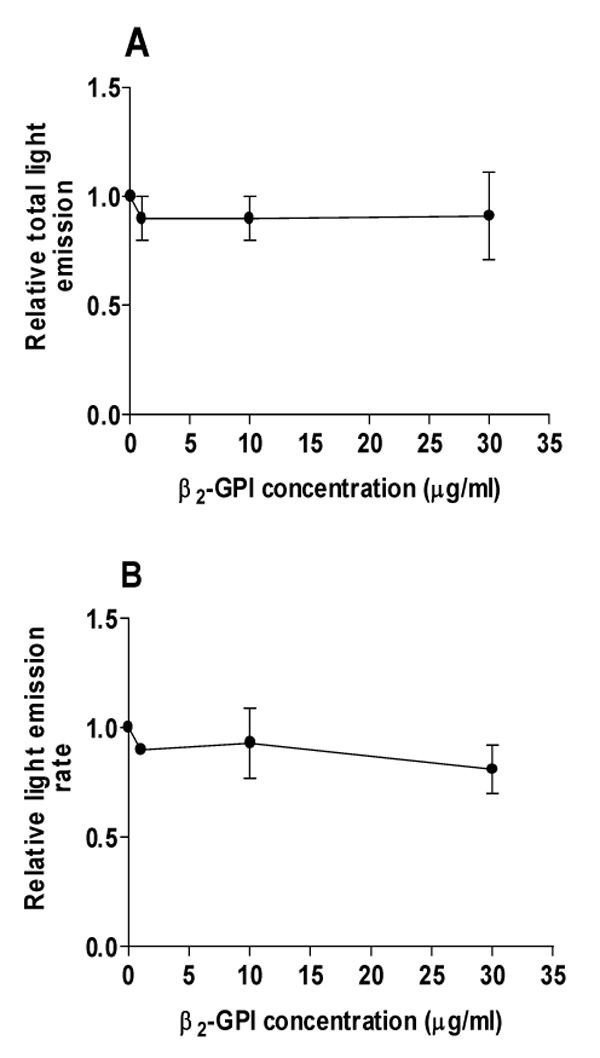
Beta_2_-glycoprotein I effects on relative (A) total light emission and (B) light emission rate of isolated cells. Means á SEM for four separate experiments.

Liver perfusion with C in the absence of beta_2_GPI led to uptake of C particles and increase in O_2 _consumption (Fig. 1, Table 1). The latter effect is mainly related to the respiratory burst of Kupffer cells [4,9], with secondary O_2 _utilization in mitochondrial respiration for energy supply needed for C phagocytosis (10) and O_2 _uptake induced in hepatocytes by eicosanoids released from activated Kupffer cells [12]. Both Kupffer cell C uptake (Fig. 1A) and C-induced O_2 _consumption (Fig. 1B) are inhibited by beta_2_GPI (1á30 micrograms/mL), with significant (p &lt; 0.05) 23% and 97% decreases being found at 30 micrograms/L beta_2_GPI, respectively. C-induced O_2 _consumption inhibition inversely correlates with beta_2_GPI concentration (r: -0.8455; p= 0.036). In agreement with biochemical data, optical microscopy revealed that C uptake by non-parenchymal cells is diminished by infusion of 30 micrograms/mL beta_2_GPI (Fig. 2). These effects by beta_2_GPI are achieved without changes in liver viability, evidenced by comparable fractional LDH effluxes among experimental groups (not shown), and are not mimicked by albumin infusion (Table I). Despite the beta_2_GPI-induced inhibition of C phagocytosis found in perfused liver, chemiluminescence of isolated non-parenchymal liver cells was insensitive to 30 micrograms beta_2_GPI/mL (Fig. 3).

**Table 1:** Carbon (C) uptake and C-induced O_2 _uptake inhibition by beta_2_-glycoprotein I in perfused mouse liver

Experimental conditions	n	C-uptake (mg/g liver/min)	C-induced O_2 _uptake (ámol/g liver)
Control	5	1.41 á 0.12	4.82 á 0.42
Beta_2_GPI (30 micrograms/mL)	4	1.08 á 0.005^a^	0.13 á 0.13^a^
Albumin (30 micrograms/mL)	4	1.30 á 0.06	4.99 á 0.53

Means á SEM for separate experiments. ^a^P &lt; 0.05 compared to control or albumin infusion, assessed by one-way ANOVA followed by the Bonferroni test.

Beta_2_GPI associates with membranes through annexins, PS receptor, lipoprotein receptors, and negatively charged phospholipids such as PS [5,13]. Interference of beta_2_GPI with PS availability in the phagocyte membranes may affect cellular responses, such as translocation of protein kinase C (PKC) to cell membranes [14]. This effect could affect PKC and subsequent triggering of PKC-dependent events, including superoxide anion generation and particle uptake [15]. From current data, it is suggested that beta_2_GPI suppresses the respiratory burst response associated with Kupffer cell phagocytosis, while discretely diminishes particle uptake. The former effect is dependent on intact liver architecture, which allows interactions among different cell-types in the liver [16].
